# Softness and weight from shape: Material properties inferred from local shape features

**DOI:** 10.1167/jov.20.6.2

**Published:** 2020-06-03

**Authors:** Filipp Schmidt, Roland W. Fleming, Matteo Valsecchi

**Affiliations:** Justus Liebig University Giessen, Germany; Center for Mind, Brain and Behavior (CMBB), University of Marburg and Justus Liebig University Giessen, Germany; Center for Mind, Brain and Behavior (CMBB), University of Marburg and Justus Liebig University Giessen, Germany; University of Bologna, Italy

**Keywords:** vision, shape perception, material perception, softness, weight

## Abstract

Object shape is an important cue to material identity and for the estimation of material properties. Shape features can affect material perception at different levels: at a microscale (surface roughness), mesoscale (textures and local object shape), or megascale (global object shape) level. Examples for local shape features include ripples in drapery, clots in viscous liquids, or spiraling creases in twisted objects. Here, we set out to test the role of such shape features on judgments of material properties softness and weight. For this, we created a large number of novel stimuli with varying surface shape features. We show that those features have distinct effects on softness and weight ratings depending on their type, as well as amplitude and frequency, for example, increasing numbers and pointedness of spikes makes objects appear harder and heavier. By also asking participants to name familiar objects, materials, and transformations they associate with our stimuli, we can show that softness and weight judgments do not merely follow from semantic associations between particular stimuli and real-world object shapes. Rather, softness and weight are estimated from surface shape, presumably based on learned heuristics about the relationship between a particular expression of surface features and material properties. In line with this, we show that correlations between perceived softness or weight and surface curvature vary depending on the type of surface feature. We conclude that local shape features have to be considered when testing the effects of shape on the perception of material properties such as softness and weight.

## Introduction

We live in a world of objects, and these objects are made out of stuff. Trees are made from wood, buildings are made from stone, many of our artifacts are made from plastic, and we ourselves are made from skin, flesh, and bones. Starting with a seminal article from Edward H. Adelson (2001), vision science and visual neuroscience got increasingly intrigued with the question of how we recognize these materials and estimate their properties (Anderson, 2011; Fleming, 2014; Fleming, 2017). For example, just by looking at them, we can figure out whether a table is made from wood or plastic (Sharan, Rosenholtz, & Adelson, 2009) and estimate the viscosity of flowing liquids (van Assen, Barla, & Fleming, 2018).

Here, we focus on the role of object shape in the estimation of mechanical material properties, specifically softness and weight. Are there certain shape characteristics that make an object appear heavy or light? Do smooth features make things look soft? Motivated by questions such as these, we sought to investigate how shape features of unfamiliar objects affect our perception of their material properties.

Previous studies showed that shape is a powerful cue for estimating material properties (Adelson, 2001; Marlow & Anderson, 2015; Pinna & Deiana, 2015; Paulun, Kawabe, Nishida, & Fleming, 2015; Schmidt, Paulun, van Assen, & Fleming, 2017; Zoeller, Lezkan, Paulun, Fleming, & Drewing, 2019), especially in combination with motion (i.e., changes in shape over time; Bi, Jin, Nienborg, & Xiao, 2018; Bouman, Xiao, Battaglia, & Freeman, 2013; Kawabe, Maruya, Fleming, & Nishida, 2015; Schmid & Doerschner, 2018; van Assen & Fleming, 2016). For example, perceived compliance is well predicted by the (perceived) deformation of object shape in short animated movies, relatively independent of optical surface properties (Fakhoury, Culmer, & Henson, 2015; Paulun, Schmidt, van Assen, & Fleming, 2017). This is also true to some extent in unfamiliar, static objects (Schmidt et al., 2017).

At the same time, shape cues to material perception operate at different scales (Koenderink & van Doorn, 1996; Sharan, Liu, Rosenholtz, & Adelson, 2013). Previous work distinguished between microscale, mesoscale, and megascale shape features (Koenderink & van Doorn, 1996), with microscale referring to microtextures (determining diffuse and specular reflection, i.e., surface roughness), mesoscale referring to visible surface textures (e.g., leathery texture), as well as local shape features (e.g., folded cloth), and megascale referring to the global shape of an object (e.g., spherical shape). Here, we focus on local shape features, which is the level at which textiles form the distinctive ripples and creases of drapery and viscous liquids clump up into clots or dollops. Also, it is the scale at which some large-scale deformations become evident, such as when an object is bent or twisted (Fleming & Schmidt, 2019; Schmidt & Fleming, 2016, Schmidt & Fleming, 2018; Schmidt, Phillips, & Fleming, 2019; Schmidt, Spröte, & Fleming, 2016; Spröte & Fleming, 2016; Spröte, Schmidt & Fleming, 2016).

**Figure 1.**
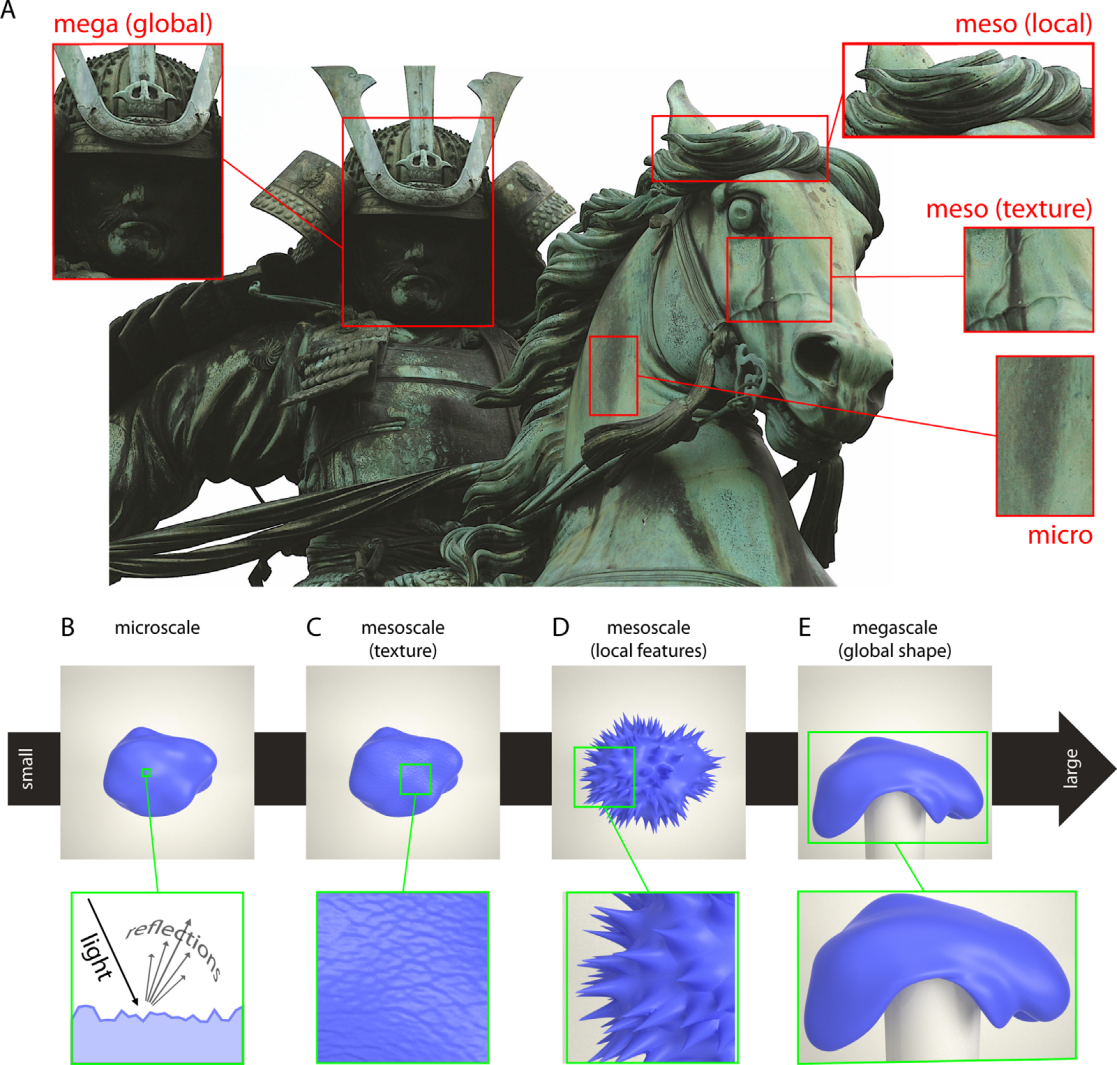
Illustration of shape cues to material perception at different scales. (A) Close-up of a statue of Kusunoki Masashige, Tokyo, in which different parts of the statue illustrate microscale (rough patina), mesoscale texture (veined horse skin), mesoscale local (floating mane), and megascale (head and helmet) shape cues to material perception (statue image copyright 2007 by Jim Epler, https://commons.wikimedia.org/wiki/File:Kusunoki_masashige.jpg, published under CC-BY 2.0; https://creativecommons.org/licenses/by/2.0/deed.en). In this study, we use computer renderings where we change the surface of a base object whose (A) microscale surface shape makes it appear glossy (and not rough). In the following, we modified the (C) mesoscale texture and (D) mesoscale local surface features, and (E) megascale global shape features (by simulating the object drooping over a static cylinder).

In Figure 1, we illustrate effects of microscale, mesoscale and megascale shape features on material perception (Figure 1A). First, on the microscale, the microscopic surface relief defines the material's bidirectional reflectance distribution function, which determines whether a material appears, for example, rough or glossy (Figure 1B; Koenderink & van Doorn, 1996). Second, on the mesoscale, we can distinguish between surface shape details on the one hand and larger, local shape features on the other. The surface shape details are equivalent to texture material features (e.g., Bhushan, Rao, & Lohse, 1997; Leung & Malik, 2001). For example, the fine details of leather material can be emulated by displacement of the object surface according to a texture image height map (Figure 1C). In contrast, local shape features are less textural with more distinct, individual forms—which also potentially signify particular material types or material properties. For example, whirls and folds for hair, textiles, or other soft materials, and angular features and spikes for hard materials (Figure 1D; e.g., Giesel & Zaidi, 2013; Pinna, 2010; Schmidt & Fleming, 2018; Schmidt et al., 2019). Fourth, on the megascale, material-related shape features are global and emerge from the interrelations between distant regions on the object or object parts. For example, a soft object might droop or bend under gravity (Figure 1E; e.g., Paulun et al., 2017; Schmidt et al., 2017). The megascale also defines the global shape of objects, such as the spherical shape of an orange (Koenderink & van Doorn, 1996). Of course, all of these scales of shape features are not qualitatively different (e.g., as the deformation magnitude of microscale shape features increases, they will eventually turn into mesoscale deformations)—but they are useful distinctions along a continuum of increasing shape deformation of object surfaces (e.g., Koenderink & van Doorn, 1996; Marlow & Anderson, 2013; Phillips, Egan, & Perry, 2009).

Microscale features are well known to affect the perception of material through their effects on light, for example with glossy objects (Chadwick & Kentridge, 2015; Cook & Torrance, 1981; Fleming, Dror & Adelson, 2003; Marlow, Kim, & Anderson, 2012; Pellacini, Ferwerda, & Greenberg, 2000).

There is also ample evidence that material recognition and estimation of material properties is affected by mesoscale texture (e.g., Bhushan et al., 1997; Heaps & Handel, 1999; Leung & Malik, 2001; Wiebel, Valsecchi, & Gegenfurtner, 2013; Wiebel, Valsecchi, & Gegenfurtner, 2014), as well as megascale (global) shape features (e.g., Bi et al., 2018; Bi & Xiao, 2016; Bouman et al., 2013; Kawabe & Nishida, 2016; Paulun et al., 2017; Schmid & Doerschner, 2018; Schmidt et al., 2017). However, the role of mesoscale local shape features has not been investigated explicitly. Here, we test the role of those shape features for material perception in novel, unfamiliar static objects. Specifically, we want to know how much local shape features contribute to judgments of material properties softness and weight; both of which are important cues to usability and behavior of objects. For example, softness is important when judging the edibility of fruits or baked goods, whereas weight is important when deciding whether objects can be easily handled or containers are empty or full. By using unfamiliar and static objects, we aim to reduce the contribution of material associations evoked by familiarity (e.g., a pillow-shaped object will be judged as soft and light) or by motion cues (e.g., a deforming object will be judged as soft and light). For the same reason, we use nonsemantic rating scales and include a control experiment to directly measure semantic associations for all of our stimuli.

## Experiments

### Experiment 1: Materials and methods

#### Participants

Fifteen students from Justus Liebig University Giessen, Germany, with normal or corrected vision participated in the experiment for financial compensation (11 women, four men, ages 19–34 years, mean 24.4 years). All participants gave informed consent, were debriefed after the experiment, and were treated according to the ethical guidelines of the American Psychological Association. All testing procedures were approved by the ethics board at Justus Liebig University Giessen and were carried out in accordance with the Code of Ethics of the World Medical Association (Declaration of Helsinki).

#### Stimuli

**Figure 2.**
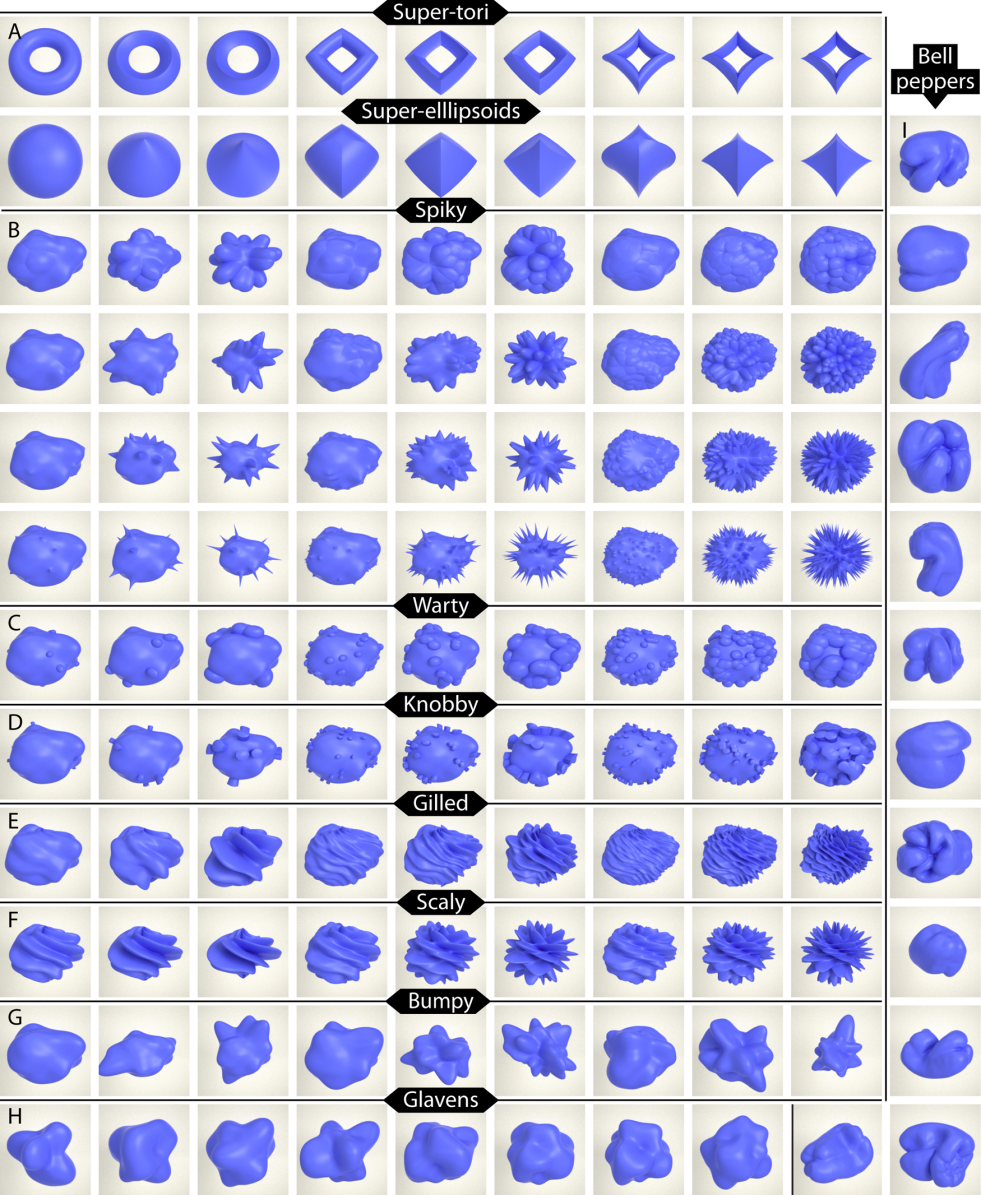
Example stimuli from all classes (organized in rows): (A) super-tori and super-ellipsoids, (B) spiky objects with four different levels of spikiness, (C) warty, (D) knobby, (E) gilled, (F) scaly, (G) and bumpy objects, as well as (H) Glavens, and (I) bell peppers (in right column). Note that this figure shows only a subset of all stimuli: super-tori and super-ellipsoids were additionally rendered from two other viewpoints, and all classes but Glavens and bell peppers contained four other base objects (each of which was perturbed according to the same function as the one base object depicted here).

The majority of stimuli were created by rendering objects created with ShapeToolbox (http://saarela.github.io/ShapeToolbox/; Saarela, 2018) for Matlab2018a (The MathWorks, Inc., Natick, MA, USA) at a resolution of 512. We started out by creating two classes of geometric objects: nine “super-ellipsoids” and nine “super-tori” (Saarela, 2018), which we rendered from three different viewpoints (adding up to 2 × 9 × 3 = 54 geometric stimuli; for examples from one of the viewpoints see Figure 2A).

Next, we created five ellipsoid base objects by randomly varying ellipsoid radii and introducing small noise perturbations. These five base objects were then subjected to different types of “transformations” (i.e., classes of surface perturbation functions), each of which contained nine members defined by all combinations of three frequency and three amplitude levels. The first four classes were variants of spiky transformations with four different levels of spikiness (adding up to 4 × 5 × 9 = 180 spiky objects) (for examples showing the four different levels of spikiness for the first base object, see rows in Figure 2B). Next, we defined two classes of protrusion transformations with warts and knobs (adding up to 2 × 5 × 9 = 90 warty and knobby objects) (for examples for the first base object, see Figures 2C and 2D) and two classes of layered transformations with gills and scales (adding up to 2 × 5 × 9 = 90 gilled and scaly objects) (for examples for the first base object, see Figures 2E and 2F). Finally, we added a class of bumpy stimuli (5 × 9 = 45 bumpy objects) (for examples for the first base object, see Figure 2G).

In addition to objects created with ShapeToolbox, we included eight Glavens (Phillips, Casella, & Egan, 2016) (Figure 2H) and 12 three-dimensional scans of bell peppers (Capsicum annuum) (Norman & Phillips, 2016) (Figure 2I) (Glavens and bell peppers are both licensed under a Creative Commons Attribution-NonCommercial-ShareAlike 4.0 International License; http://creativecommons.org/licenses/by-nc-sa/4.0/). Both types of stimuli were used in a number of previous studies (e.g., Dövencioglu, van Doorn, Koenderink, & Doerschner, 2018; Ennis & Doerschner, 2019; Norman, Phillips, Cheeseman, Thomason, Ronning, Behari, Kleinman, Calloway, & Lamirande, 2016; Norman, Norman, Clayton, Lianekhammy, & Zielke, 2004; Phillips et al., 2009). Altogether, our set comprised 479 stimuli.

Finally, we loaded each object into RealFlow10 (NextLimit Technologies, Madrid, Spain) and assigned them a blue plastic material that in previous studies was found to be perceived as having intermediate softness (Paulun et al., 2017; Schmidt et al., 2017). We placed the object in front of a white wall and rendered the scene in Maxwell (NextLimit Technologies, Madrid, Spain) using a studio-like environment map. The sampling level was 15, and image resolution was 300 × 300 pixels (all stimuli can be obtained from https://doi.org/10.5281/zenodo.3816665).

#### Rating scale

**Figure 3.**
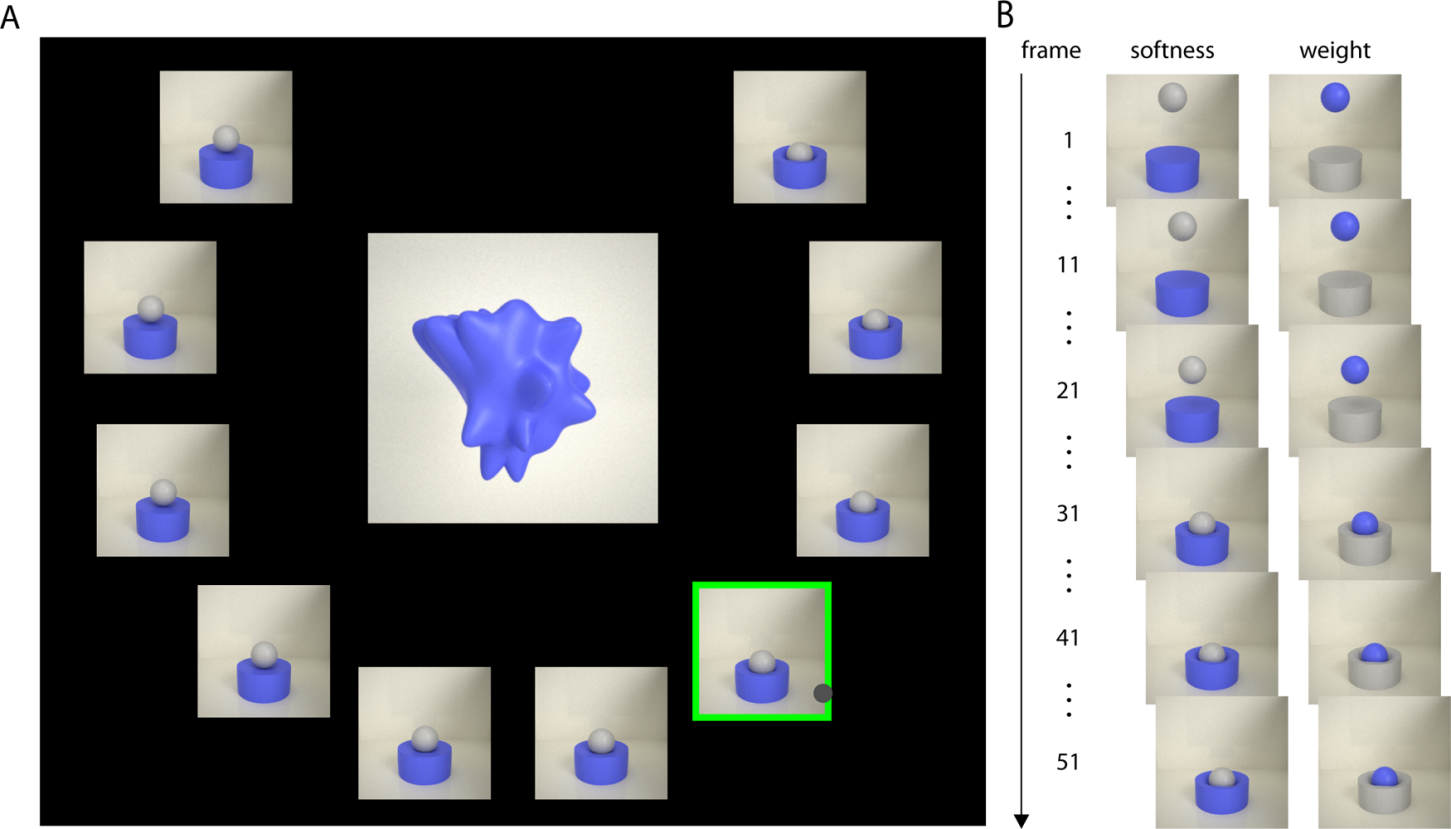
Rating paradigm. (A) Example trial of Experiment 1 (softness rating). The stimulus is presented at the center of the screen surrounded by the animation sequences in a horseshoe arrangement and playing in a loop. Here, for demonstration purposes, we show the last frame of each animation. Participants picked the animation which behaved as they would expect if it would stem from the central object by selecting it via mouse click (grey disc; animations the mouse is hovering over are marked by a green frame). (B) Example frames of animation sequences for the “softest” and “heaviest” animation sequences. Note that in the softness animations the relevant material sample is the (blue) cylinder, while in the weight animations it is the (blue) ball.

Many previous material perception studies have used rating tasks in which participants are presented with a word for a material property (e.g., “softness”)—possibly accompanied with a verbal definition of the term—and are asked to report the extent to which each stimulus exhibits this characteristic (Fleming, Wiebel, & Gegenfurtner, 2013; Kawabe & Nishida, 2016; Kawabe, Maruya, & Nishida, 2015; Paulun et al., 2017; Sawayama, Adelson, & Nishida, 2017; Schmid & Doerschner, 2018; Schmidt et al., 2017; van Assen et al., 2018; van Assen & Fleming, 2016). Although this approach has delivered many useful results, it can suffer from a degree of subjectivity in the interpretation of the verbal term and instructions, leading to variability between participants. Here, instead, we used a visual scale in which we presented participants with a set of 10 animations depicting different softness values, and they identified which one best indicated the apparent softness of the test stimulus. For this, we created a simple scene in RealFlow10 (NextLimit Technologies, Madrid, Spain) consisting of a ball falling onto a shallow cylinder of deformable material with different stiffness levels (Figure 3). The cylinder was defined as a soft body with a resolution of 125, its elasticity was set to 0.0 (on a scale from 0.0 to 1.0, describing the amount of energy that is retained by the body when it collides); internal damping was set to 50.0 (which influences the time after which the object stops bouncing, as well as the size of the bounces); and friction was set to 1.0 (on a scale from 0.0 to 1.0). We varied length stiffness and volume stiffness, the recovery constants relative to the object that determine the resistance of the object against changes in its original volume or its longitudinal magnitudes, respectively (Realflow10 allows variations on a scale from 0.0 to 1000.0). We varied both parameters simultaneously in ten steps from “hard” to “soft” appearance: 6.561, 2.187, 0.729, 0.243, 0.081, 0.027, 0.009, 0.003, 0.001, and 0.001 (the final one plus reduced internal damping of 35.0). These values are nondimensional and have no direct analogue in physics so that we will refer to the different levels on an ordinal scale from 1 to 10 (with 10 being the softest). To the best of our knowledge, to this day there is no simulation engine for soft bodies that is physically accurate across such a wide range of softness levels and object shapes as tested here. However, the RealFlow10 simulations yield visually compelling impressions of realistic deformable materials with different stiffness and are approximately perceptually uniform. Finally, we assigned the same blue material to the cylinder as we previously assigned to the stimulus objects. Then, we rendered 51 frames of the resulting animations in Maxwell (NextLimit Technologies, Madrid, Spain) using a studio-like environment map. The sampling level was 15 and image resolution was 300 × 300 pixels (all rating animations can be obtained from https://doi.org/10.5281/zenodo.3816665). In the remainder of the article, we refer to ratings on this scale as softness ratings.

#### Procedure

In each trial, we presented participants with one of the test stimuli at the center of the screen. The animations of the rating scale were presented in a horseshoe arrangement around the center and played in loop (Figure 3). We instructed participants as follows: “Your task is to estimate the material properties of the static object presented at the centre of the screen. Surrounding this object you see repeating animation sequences in which a ball is falling onto a material sample. In each trial, choose that sample which is behaving as you would expect it to, if it were made of the same material as the central object.” Each participant responded to each of the 479 test stimuli in random order, selecting the corresponding animation sequence by clicking on it.

Stimuli were presented on a black background on a EIZO CG277 monitor at a resolution of 2560 × 1440 pixels and a monitor refresh rate of 59 Hz, controlled by Matlab2018a (The MathWorks, Inc., Natick, Massachusetts, United States) using the Psychophysics Toolbox extension (Kleiner, Brainard, & Pelli, 2007). The height and width of each stimulus on screen was 10.6 × 10.6 cm (about 12.15° × 12.15° of visual angle at a monitor distance of about 50 cm); the height and width of each rating animation was about 4.6 × 4.6 cm (5.27° × 5.27°). The distance between the center of the stimulus and the centers of the rating animations was about 13.5 cm (15.47°).

### Experiment 2: Materials and methods

#### Participants

Fifteen students from Justus Liebig University Giessen, Germany, with normal or corrected vision participated in the experiment for financial compensation (nine females, six males, ages 20–36 years, mean 24.8 years). All other details and participant procedures were the same as in Experiment 1.

#### Stimuli

Stimuli were the same as in Experiment 1.

#### Rating scale

The creation of the animation sequences for the rating scale for weight was analogous to Experiment 1. Again, we created a simple scene with a ball falling onto a cylinder, but this time the ball had varying weight. The cylinder was equivalent to the softest cylinder from Experiment 1, and we varied the mass of the ball in ten steps from “light” to “heavy” appearance: 0.01, 2.5, 5.0, 10.0, 30.0, 60.0, 90.0, 120.0, and 150.0. Again, we will refer to the levels on an ordinal scale from 1 to 10 (with 10 being the heaviest). Finally, we assigned the same blue material to the ball as we previously assigned to the stimulus objects and rendered the resulting animations in Maxwell (NextLimit Technologies, Madrid, Spain) with the same details as before (all rating animations can be obtained from *https://doi.org/10.5281/zenodo.3816665*). In the remainder of the article, we refer to ratings on this scale as weight ratings.

#### Procedure

The procedure of the weight rating task was equivalent to the softness rating task. However, after the rating participants also completed a free naming task. Here, they were asked whether the *shape* of a stimulus looked like (a) a particular object (e.g., chair, cup); (b) it was made from a particular material (e.g., wood, water); (c) it was produced by a particular transformation (e.g., bent, folded); or (d) none of those. If they responded yes to (a) to (c), they were asked to type in the object, material, or transformation. All stimuli from the previous experiments were presented in random order, with each participant responding to about 160 stimuli—yielding five free-naming responses per stimulus.

#### Analysis

Because the mapping of numbers to rating scale animations is arbitrary, and we expected stimuli that are perceived as softer to be also perceived as lighter rather than heavier (and vice versa for harder stimuli), we inverted the weight rating scale for all analyses. With this inverted weight scale, lower values denote heavier and higher values denote lighter ratings.

### Results

#### Rating results

**Figure 4.**
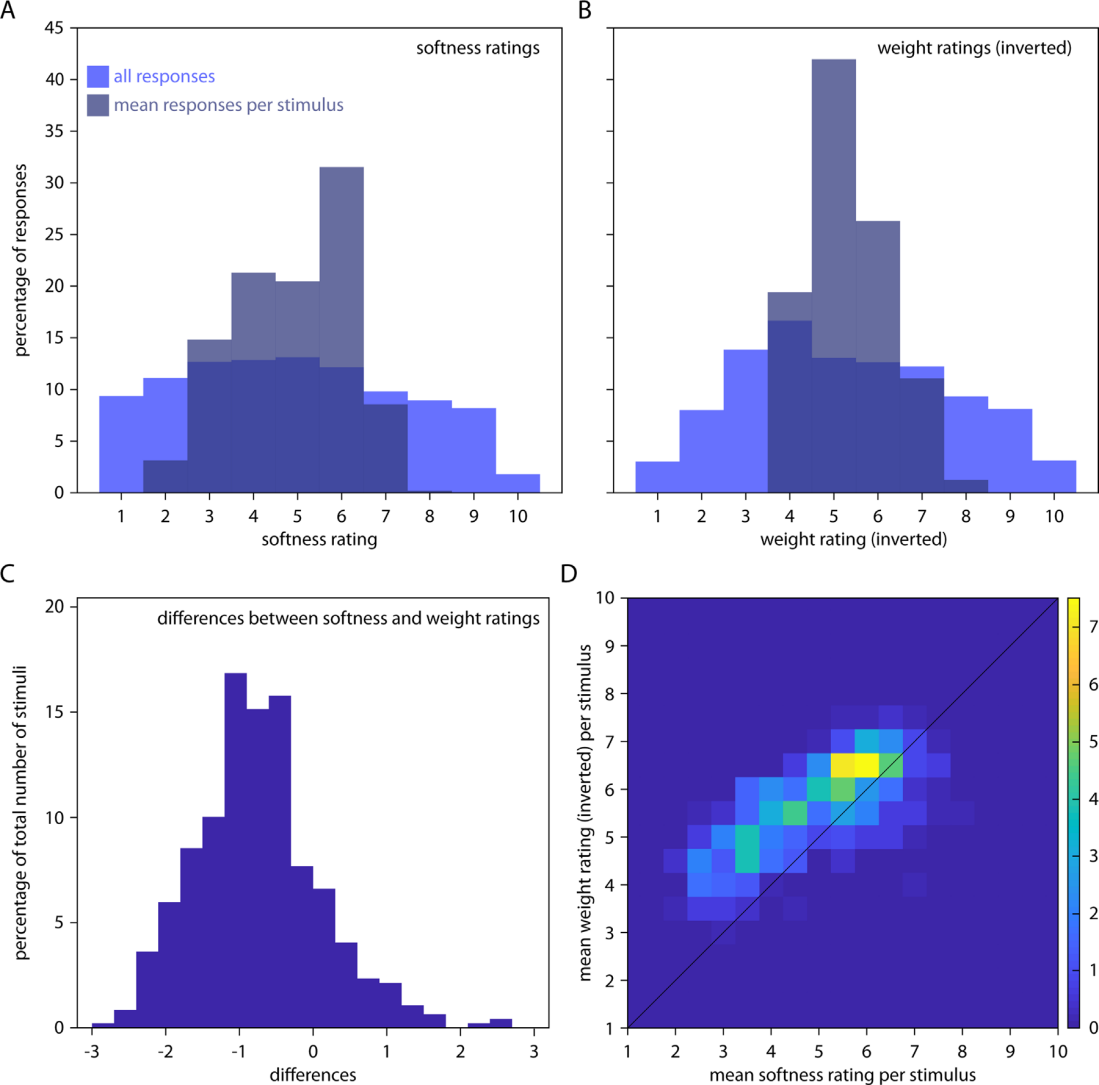
Softness and weight ratings and their relationship. Distribution of (A) softness and (B) inverted weight ratings for all responses (light blue) and responses averaged per stimulus (dark transparent blue), expressed as percentage of responses. The grand mean across participants and stimuli for softness ratings was 4.91 (SD = 2.49) and for weight ratings was 5.33 (SD = 2.31). (C) Distribution of differences between average ratings of softness and inverted weight ratings, calculated per stimulus and expressed as percentage of the total number of stimuli. (D) Heat map showing the distribution of all 479 stimuli in the space spanned by softness and inverted weight ratings, with the color bar referring to the number of responses in percent. The correlation coefficient between ratings is r = 0.75 (R^2^ = 0.56).

First, we tested whether we obtained any effects of stimulus shape on perceived softness and weight (all rating data can be obtained from https://doi.org/10.5281/zenodo.3816665). Our results show that softness and weight ratings both spanned a wide range of the respective rating scales: after averaging across participants, the minimum and maximum softness ratings obtained for any stimuli were 2.2 and 7.8 (Figure 4A), and the minimum and maximum weight ratings obtained for any stimuli were 3.13 and 7.4 (Figure 4B). This suggests that our stimuli were suitable to evoke a wide range of different softness and weight responses.

Second, we looked at how well participants agreed with each other by calculating mean correlation coefficients between ratings of all participants per stimulus, resulting in a correlation of *r* = 0.26 for softness ratings, and *r* = 0.21 for weight ratings. As a more formal measure, we also calculated intraclass correlation (*ICC*; McGraw & Wong, 1996). ICC measures the reliability of ratings and can take values between 0 (low similarity between ratings) and 1 (high similarity between ratings). We report ICC(C,1) for consistency between ratings (i.e., relative agreement) and ICC(A,1) for absolute agreement between ratings (McGraw & Wong, 1996). For softness ratings, we obtained ICC(C,1) = 0.28 and ICC(A,1) = 0.22, both of which were significantly different from zero (F[478, 311.49] = 6.71, *p* < 0.001, and F[478, 6692] = 6.71, *p* < 0.001, respectively). For weight ratings, we obtained ICC(C,1) = 0.12 and ICC(A,1) = 0.10, both of which were significantly different from zero (F[478, 6692] = 3.08, *p* < 0.001, and F[478, 766.09] = 3.08, *p* < 0.001, respectively). Together, this shows that even though there was substantial variation among participants, they also agreed with each other in their softness and weight ratings above chance—showing that there was truly some effect of shape on perceived softness and weight. At the same time, there was less agreement among participants for weight ratings, suggesting that softness from shape was more immediate to participants compared to weight from shape.

Third, we looked at how much softness and weight ratings agreed with each other. At the level of individual stimuli, ratings were clearly different (Figure 4C)—showing that participants were not just associating any salient change in surface to some change in material levels. At the same time, there was considerable correlation between the two ratings (*r* = 0.75, *R*² = 0.56), such that stimuli that were rated as softer also tended to be rated as lighter, and harder stimuli tended to be rated as heavier (Figure 4D). Part of this correlation might result from the similarities between the two visual rating scales, but part of the correlation likely also results from correlations between stiffness and density in the natural world (Ashby, 2013). However, because these correlations vary strongly depending on the tested materials and we do not have a mapping between our stimuli and real materials and objects, we cannot determine the relative contribution of these two explanations to our findings.

In the following, we took a closer look at the results for each class of stimuli, starting with super-tori and super-ellipsoids, followed by the spiky, warty, knobby, gilled, scaly, and bumpy objects, and concluding with the Glavens and bell pepper stimuli.

**Figure 5.**
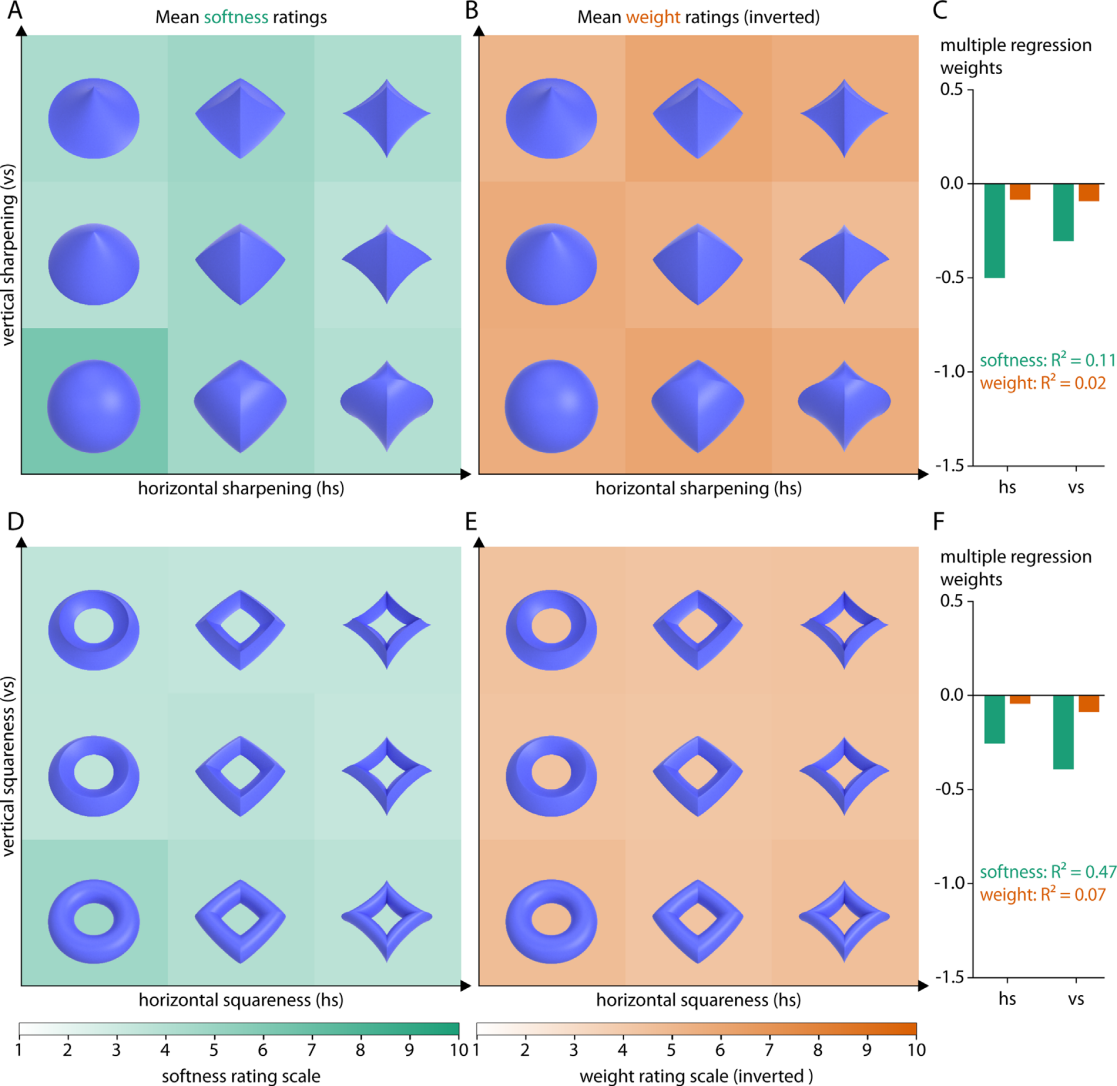
Rating results for geometric objects. (A, D) Softness ratings and (B, E) inverted weight ratings for super-ellipsoids and super-tori, respectively, averaged across participants and the three viewpoints. More saturated green values correspond to softer objects, more saturated orange values correspond to lighter objects. Stimuli are plotted as a function of the two parameters defining their horizontal sharpening/squareness and vertical sharpening/squareness (see text for details). (C, F) Multiple regression fits (R^2^ values) and regression weights for the two parameters on mean softness (green) or mean inverted weight (orange) ratings.

Figure 5 shows rating results for super-ellipsoids (5A–5C) and super-tori (5D–5F). Stimuli are plotted as a function of the two parameters defining their horizontal and their vertical sharpening/squareness. Mean softness ratings of super-ellipsoids were about M = 3.9 (range 2.2–7.2; standard deviation [SD] = 1.49), mean weight ratings were about M = 5.1 (range 3.9–7.0; SD = 0.80). Mean softness ratings of super-tori were about M = 3.0 (range 2.2–4.7; SD = 0.57), mean weight ratings were about M = 3.8 (range 3.1–4.4; SD = 0.32). Note that here and in the following, standard deviation (SD) always refers to rating differences between objects of the set rather than between participants. Within classes, only softness ratings for the most rounded objects (i.e., for the sphere in the super-ellipsoids and the torus in the super-tori; lower left stimuli in Figures 5A and 5D) stand out; weight ratings were much less systematic. In line with this, multiple regression analyses using horizontal and vertical sharpening/squareness as factors only explained a substantial proportion of the variance in softness ratings for super-tori (*R*² = 0.47) but not for softness ratings for super-ellipsoids (*R*² = 0.11) or weight ratings (super-ellipsoids: *R*² = 0.02; super-tori: *R*² = 0.07).

**Figure 6.**
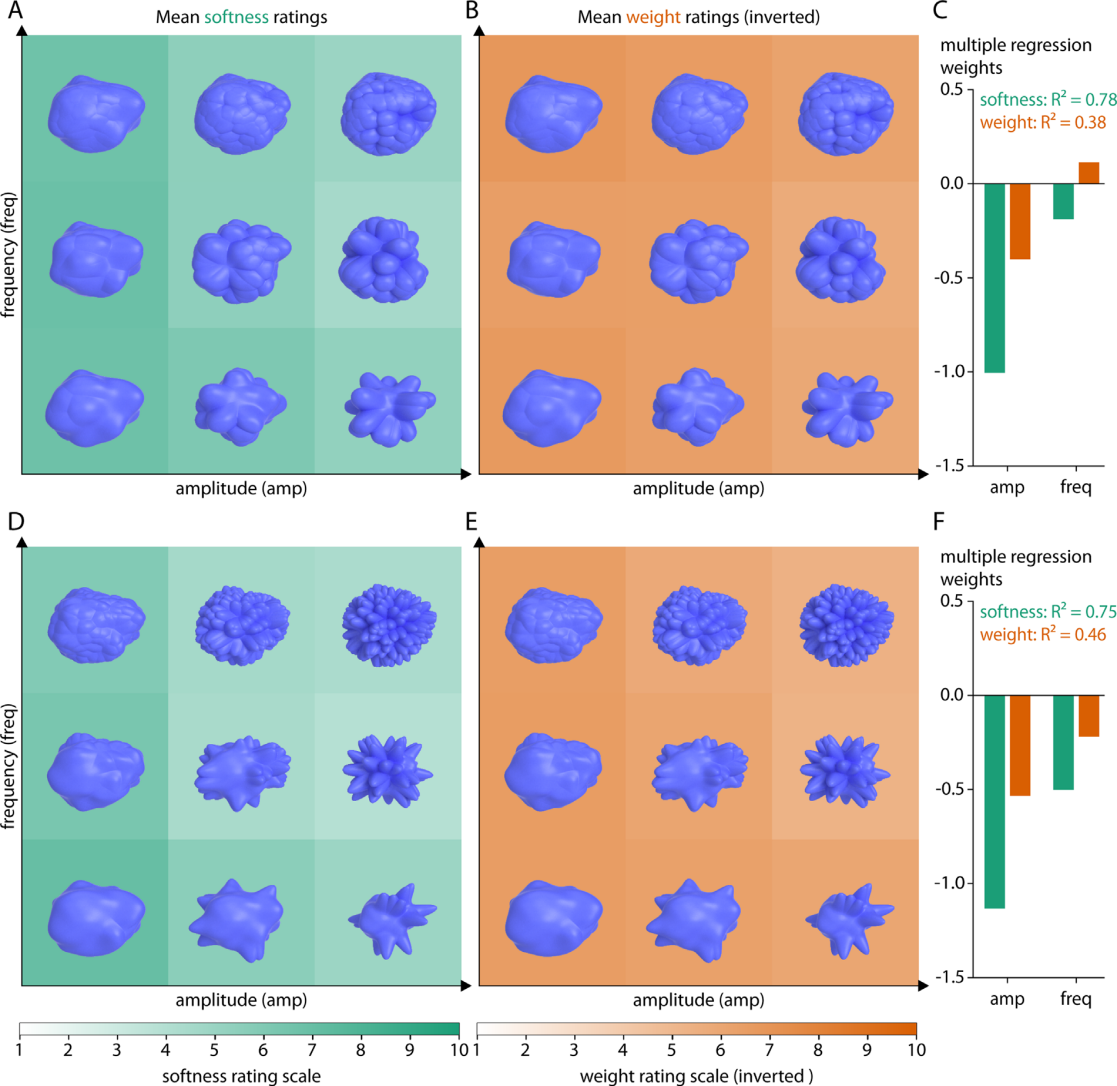
Rating results for two classes of relatively blunt spiky objects. (A, D) Softness ratings and (B, E) inverted weight ratings for two different levels of spikiness, respectively, averaged across participants and the five base objects. More saturated green values correspond to softer objects, more saturated orange values correspond to lighter objects. Stimuli are plotted as a function of spike amplitude and frequency (see text for details). (C, F) Multiple regression fits (R^2^ values) and regression weights for amplitude and frequency on mean softness (green) or mean inverted weight (orange) ratings.

**Figure 7.**
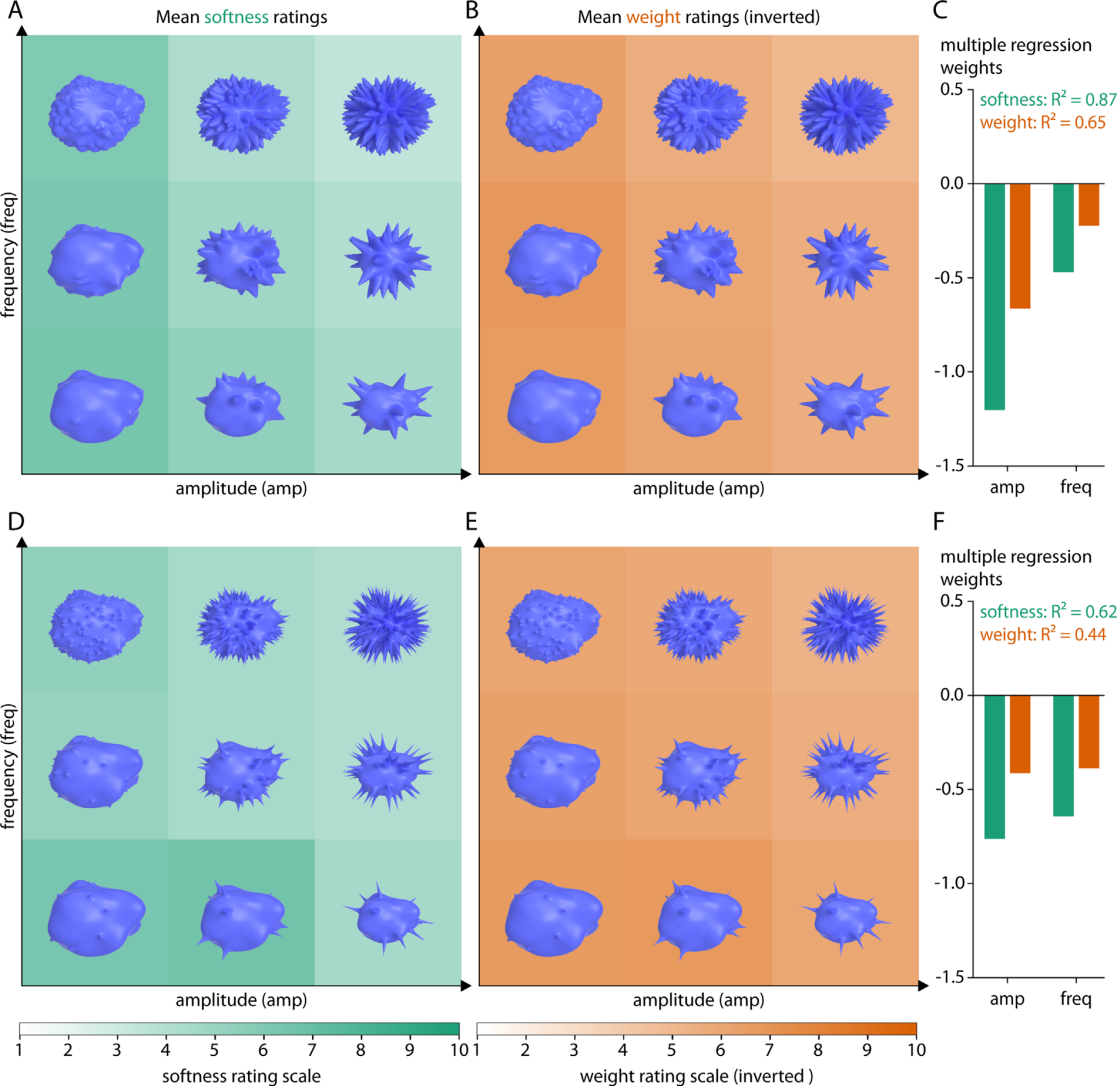
Rating results for two classes of relatively pointed spiky objects. (A, D) Softness ratings and (B, E) inverted weight ratings for two different levels of spikiness, respectively, averaged across participants and the 5 base objects. More saturated green values correspond to softer objects, more saturated orange values correspond to lighter objects. Stimuli are plotted as a function of spike amplitude and frequency (see text for details). (C, F) Multiple regression fits (R^2^ values) and regression weights for amplitude and frequency on mean softness (green) or mean inverted weight (orange) ratings.

Figures 6 and 7 show rating results for spiky objects of different spikiness. Stimuli are plotted as a function of the two parameters defining the amplitude and frequency of the spikes. Across all spiky stimuli, the mean softness ratings with increasing spikiness were M = 5.3, 4.7, 4.5, and 4.5 (range 3.5–7.3, 2.9–7.5, 2.6–6.9, 2.9–6.5; SD = 0.96, 1.18, 1.14, and 1.04): objects were judged as harder with increasing spikiness (Figures 6A and 6D; Figures 7A and 7D). The mean weight ratings with increasing spikiness were M = 6.0, 5.6, 5.5, and 5.6 (range 4.7–6.9, 3.9–6.8, 4.1–7.0, 4.4–7.3; SD = 0.56, 0.71, 0.72, and 0.70): objects were also judged as somewhat heavier with increasing spikiness (Figures 6B and 6E and Figures 7B and 7E). Equivalently within each class, increasing amplitude and frequency of spikes was correlated with judgments as being harder and heavier, with a stronger effect on softness ratings. In line with this, multiple regression analyses using spike amplitude and frequency as factors explain different amounts of variance for softness ratings (*R*² = 0.78, 0.75, 0.87, and 0.62) as for weight ratings (*R*² = 0.38, 0.46, 0.65, and 0.44) (Figures 6C and 6F and Figures 7C and 7F).

**Figure 8.**
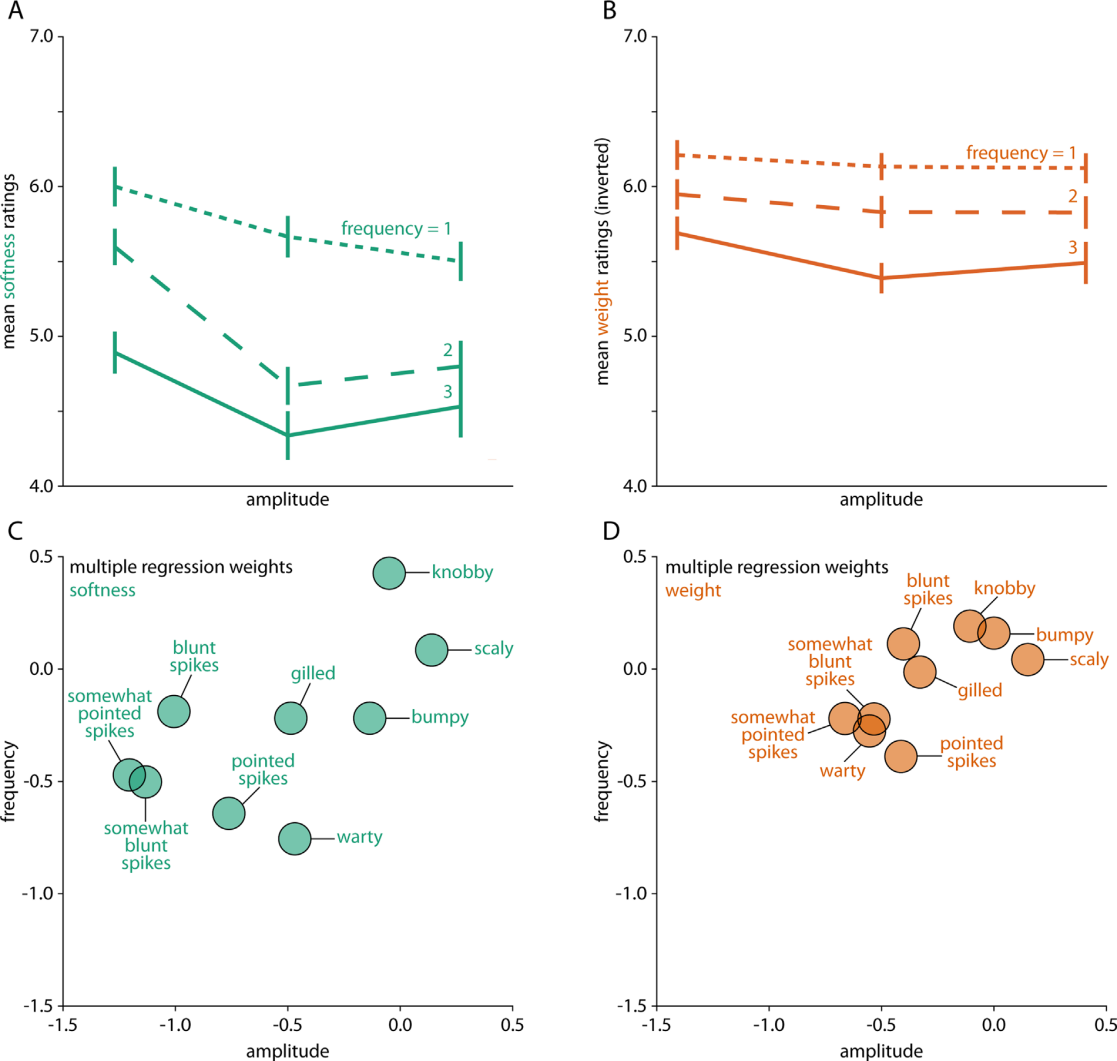
Average results across spiky, protrusion, layered and bumpy stimuli for (A) softness ratings and (B) inverted weight ratings, with the frequencies of surface shape features (different lines) plotted as a function of their amplitude. Lower panels show multiple regression weights for (C) softness ratings and (D) inverted weight ratings, plotted as a function of amplitude and frequency of surface shape features and separately for each stimulus class.

In Figure 8, we show overall results including not only spiky (four classes), but also protrusion (two classes), layered (two classes), and bumpy (one class) stimuli (405 stimuli in total). These were all classes of objects where we varied the amplitude and frequency of the shape surface features—allowing us to test the effect of these parameters on perceived softness and weight. For the sake of conciseness, we plot average softness and weight ratings across stimuli of all classes; for detailed results for protrusion, layered and bumpy stimuli refer to Supplementary Figures S1 to S3. As can be seen from the subtle but systematic diagonal gradient in Figures 8A and 8B, overall, mean softness and (inverted) weight ratings decrease with higher amplitude and frequency, showing that objects with larger and more surface shape features (e.g., spikes, warts, gills) were judged as harder and heavier compared to objects with smaller and fewer surface deformations. Multiple regression analyses using surface shape feature amplitude and frequency as factors explain different amounts of variance for the nine classes for softness (Figure 8C) and weight ratings (Figure 8D)—with generally smaller effects of amplitude and frequency on weight ratings.

**Figure 9.**
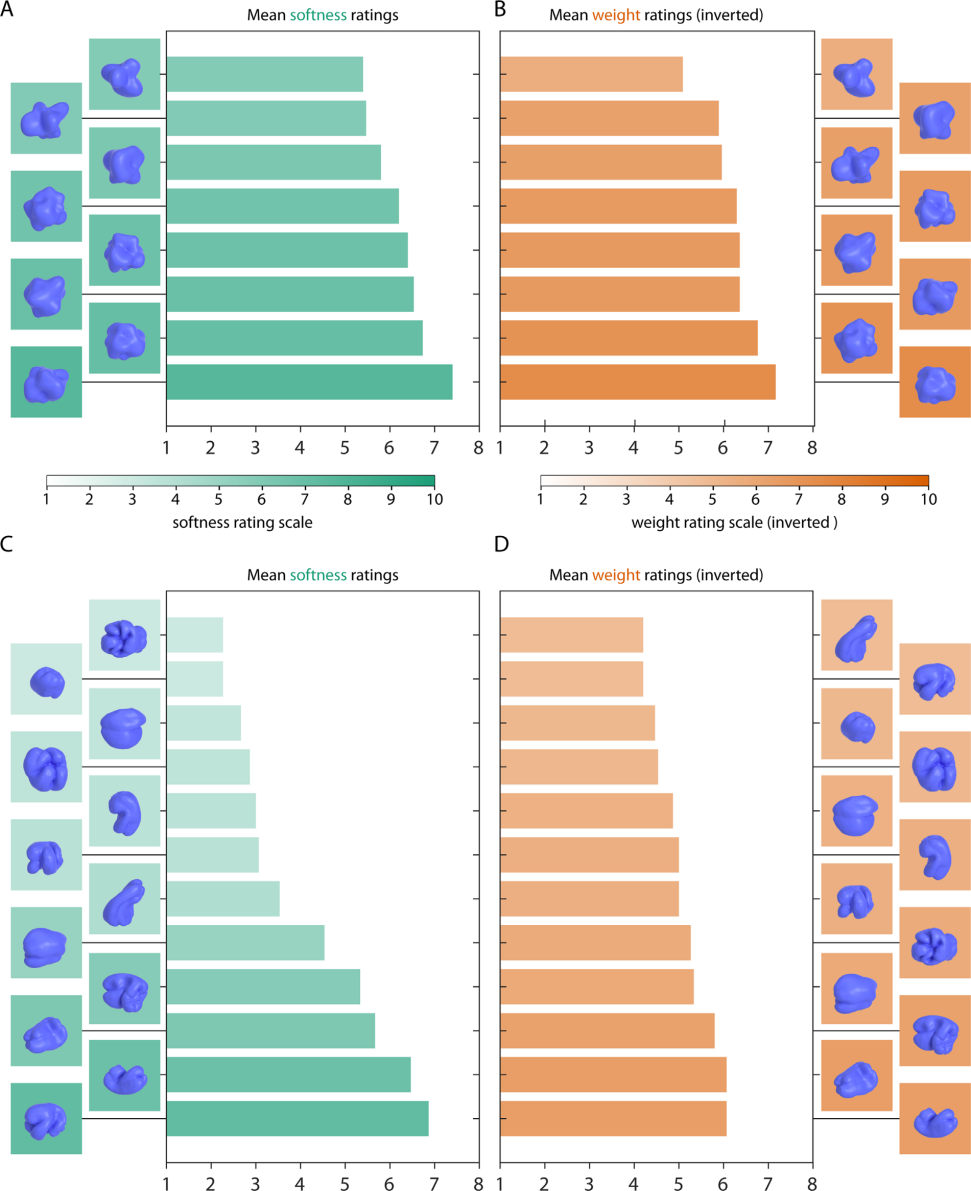
Rating results for the Glavens and bell peppers. (A, C) Softness ratings and (B, D) inverted weight ratings for Glavens and bell peppers, respectively, averaged across participants. More saturated green values correspond to softer objects, more saturated orange values correspond to lighter objects. Stimuli are plotted in the order of their average softness and weight ratings (with softer and lighter stimuli at the top).

Finally, Figure 9 shows rating results for Glavens (A–B) and bell peppers (C–D). Mean softness ratings of Glavens were about M = 6.2 (range 5.4–7.4; SD = 0.68), mean weight ratings were about M = 4.8 (range 3.9–5.9; SD = 0.62). Mean softness ratings for bell peppers were about M = 4.0 (range 2.3–6.9; SD = 1.66), mean weight ratings were about M = 5.9 (range 4.9–6.8; SD = 0.66). Although average softness and weight ratings did not vary too much among different Glavens, there were strong differences among softness ratings of different bell peppers.

To relate softness and weight perception more directly to differences in object shape, we calculated the local 3D surface curvatures from the object's 3D meshes as a basic measure of shape characteristics. To do this, we used the *Graph theory toolbox* (https://www.mathworks.com/matlabcentral/fileexchange/5355-toolbox-graph/; Peyre, 2009) for Matlab2018a (The MathWorks, Inc., Natick, MA, USA) with *curvature smoothing* set to 100. At this level of smoothing, the measure is susceptible to large rather than small changes in surface orientation. We obtained principal curvatures k_1_ and k_2_ for each point (vertex) on the surface, describing the maximum and minimum of surface bends at that point. Then we compute the *mean curvature = abs(*k_1_*)+abs(*k_2_*)* per vertex. We calculate a single curvature estimate per object by averaging the mean curvature across all of its visible vertices (i.e., only considering those vertices that were visible from the camera when rendering the stimuli from the mesh objects).

**Figure 10.**
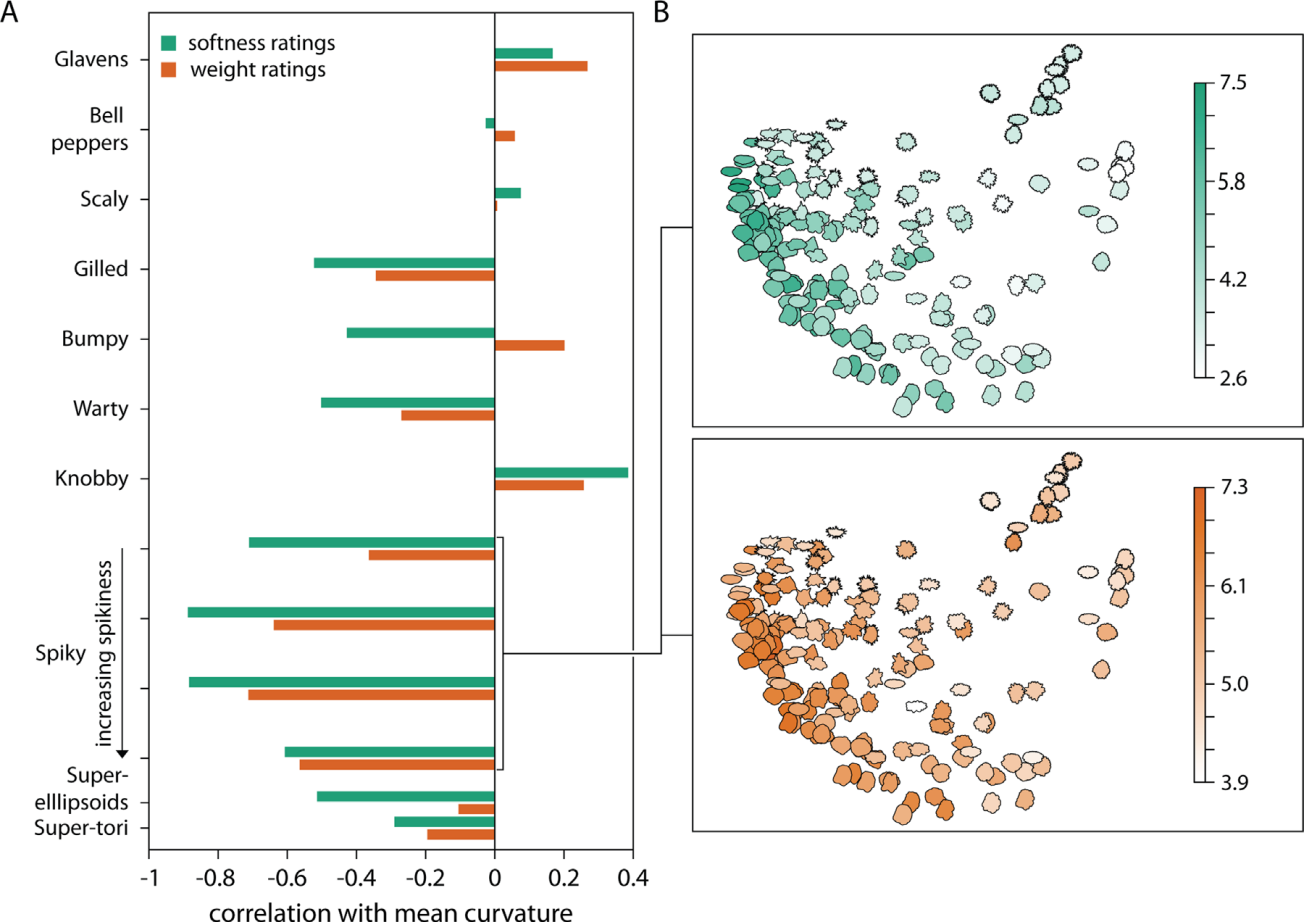
Relationship between surface curvature and softness/weight ratings. (A) Correlation coefficients between mean curvature and average softness (green) and weight (orange) ratings separately for each class of stimuli. (B) Multidimensional scaling visualization of differences in surface curvature for a single class (most pointed spiky objects), colored according to their average softness (upper panel) and weight ratings (lower panel). The first two dimensions explain about 95% of variance. The color range is normalized to the range obtained for this stimulus class (cf. color bars).

When correlating this measure of surface curvature with the average softness and weight ratings per object within each class, we obtain substantial correlations for many of the classes (Figure 10A), whereas correlations are rather low across classes (softness: *r* = −0.23, *p* < 0.001; weight: *r* = −0.11, *p* = 0.019). To visualize the stimulus organization by curvature within classes, we summarized the curvature distributions of each object in 100-bin histograms of mean curvatures and then computed the absolute difference between the histograms (by averaging the absolute differences between each of the 100 bins) for all object pairs within a class, yielding a dissimilarity matrix. As an example, we visualize the organization by surface curvatures for the class of spiky objects by using multidimensional scaling (Figure 10B). Clearly, softness and weight ratings form local clusters in the resulting space: objects with more and pointier spikes are judged as harder and heavier.

This suggests that judgments of material properties are based on surface shape features: for a number of object classes (e.g., spiky, knobby, gilled, bumpy, warty objects), participants’ responses are in accordance with a strategy relying to some extent on curvature as a measure of surface variation. However, this is not true for other classes, either because of low variance in responses between individual stimuli (also indicated by low weights in the multiple regressions, e.g., for scaly objects), of low variance in mean curvature between individual stimuli (e.g., for Glavens), or for other, more complicated, reasons (e.g., for bell peppers).

#### Free naming results

Even though we use novel, broadly unfamiliar objects, there is a chance that local shape features affected material property judgments because our objects were associated with particular familiar objects and their particular material properties (i.e., a pillow-shaped object will be judged as soft and light). For example, bell peppers are potentially recognizable, as are many of the others to a lesser extent. Similarly, the shape of our objects could directly trigger associations with particular material appearances (i.e., a surface with a leathery texture will be judged as soft), or it could be associated with particular material properties via inferred transformations (i.e., a twisted object will be judged as soft; see Discussion).

**Figure 11.**
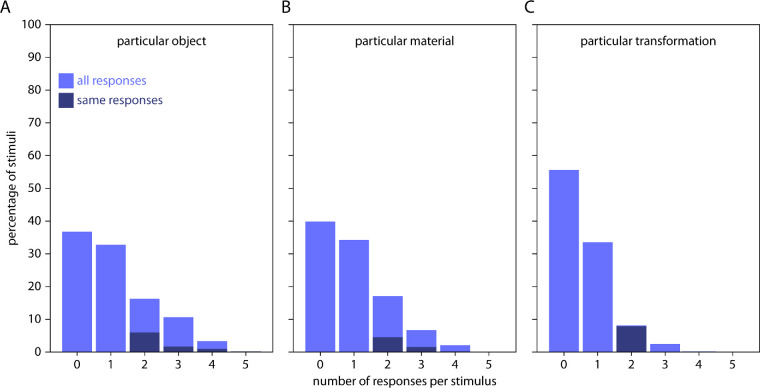
Percentage of stimuli that participants associated with a particular (A) object, (B) material, or (C) transformation, plotted for each of the possible number of responses (zero to five responses from the five participants). Light blue bars indicate percentage of all responses, dark blue bars indicate percentages of same responses.

To test empirically whether our stimuli were reminiscent of particular objects, materials or transformations, each stimulus was responded to by five participants (all naming data can be obtained from https://doi.org/10.5281/zenodo.3816665). For many stimuli, not a single participant had any association (37%, 40% and 56% for object, material, and transformation responses, respectively; Figure 11). Also, even for stimuli where at least two participants (out of five) provided a response, agreement between participants was very low. For example, only for 6% of stimuli, two participants agreed on the same particular object (Figure 11A). At the same time, agreement between more than two participants was negligible (i.e., 1.7% or lower). These values are similar for materials (two participants: 4.5%; more than two: 1.6% or lower; Figure 11B) and transformations (two participants: 7.9%; more than two: 0%; Figure 11C). Across all stimuli, the five most frequent object responses were *flower* (with only 1.7% of responses), *brain* (1.4%), *spinning top* (0.9%), *organ* (0.9%), and *blossom* (0.8%) (Supplementary Figure S4A); the five most frequent material responses were *plastic* (2.4%), *putty* (2.2%), *wax* (1.7%), *water* (1.6%), and *stone* (1.6%) (Supplementary Figure S4B); the five most frequent transformation responses were *bent* (1.1%), *folded* (1.0%), *squeezed* (0.9%), *twisted* (0.6%), and *sharpened* (0.5%) (Supplementary Figure S4C).

Overall, this indicates that our stimuli were not clearly associated with particular objects, materials or transformations, so that semantic associations most likely do not explain our findings. Specifically, as few participants saw something particular in our stimuli, and even those that did hardly agreed with the other participants, softness and weight ratings result from direct estimations of material properties from shape features—rather than from associations of object shape with familiar objects, materials, or transformations (see Discussion).

## Discussion

Material perception is an emerging field in vision science and visual neuroscience concerned with the visual recognition of materials and estimation of their properties (Anderson, 2011; Fleming, 2014; Fleming, 2017). The shape of objects and surfaces is an important cue for material perception and operates at different spatial scales: microscale (surface roughness), mesoscale (textures or local object shape), or megascale (global object shape).

Here, we investigate mesoscale local shape features between textural and global shape cues. For example, localized surface deformations (such as indentations, cuts or spikes) are clearly at a larger scale than surface textures but not necessarily integral part of global object shape (e.g., think of an indented bumper bar). This article is thus closely related to investigations of localized transformations of two-dimensional contour or three-dimensional object shape (Fleming & Schmidt, 2019; Kubilius, Bracci, & Op de Beeck, 2016; Op de Beeck, Torfs, & Wagemans, 2008; Pinna, 2010; Pinna & Deiana, 2015; Schmidt et al., 2019). These studies showed that observers can identify transformed regions of objects, and use them to make inferences about object classes, causal history, or material identity. Here, we set out to investigate the role of local shape features on judgments of material properties softness and weight. We tested 479 novel stimuli specifically designed to exhibit a wide range of different (surface) shape feature types and magnitudes, and we used animated, nonsemantic rating scales to reduce the contribution of semantic associations.

Across all stimuli, we find a substantial correlation between softness and weight ratings. Generally, stimuli that are rated as softer also tend to be rated as lighter, and harder stimuli tend to be rated as heavier. This replicates earlier findings (Schmid & Doerschner, 2018) and might reflect probabilistic heuristics about our environment, in which light objects tend to be softer (i.e., can be deformed easily) because they are very thin (e.g., textiles, paper), hollow/filled with light stuffing (e.g., soccer balls, pillows), or their material is less dense (Ashby, 2013).

At the same time, softness and weight ratings are also clearly different at the level of individual stimuli, showing that participants are not just associating any salient change in surface to some change in material levels. This is corroborated by the fact that agreement between participants was higher for softness compared to weight ratings (Schmid & Doerschner, 2018), indicating that softness from shape is more immediate to participants compared to weight from shape.

Finally, for many of the object classes studied here, judgments of material properties correlate somewhat with surface curvature measured from object meshes. This suggests that relatively straightforward measures of surface variation might play an important role in determining the expected weight or softness of unfamiliar objects. At the same time, curvature explains responses to a different degree for different object classes, indicating that participants rely on curvature to a different extent for different types of surface features. In the following, we will discuss findings for the different types of stimuli and implications for studies on material perception.

First, as comparison and control, we included stimuli varying at a megascale shape level: geometric objects (Figures 2A and 5), Glavens (Figures 3H and 7) and bell peppers (Figures 3I and 7). In line with previous findings, we find an effect of global object shape (deformations) on softness ratings (Han & Keyser, 2015; Paulun et al., 2017; Pinna & Deiana, 2015; Schmid & Doerschner, 2018; Schmidt et al., 2017). Specifically, the most rounded geometric objects seem to be judged as softer (and slightly lighter), the more angular objects (with higher levels of sharpening and squareness) are judged as harder (and slightly heavier)—with a stronger effect in the class of super-ellipsoids (Figures 5A and 5B) compared with super-tori (Figures 5D and 5E). Also, super-tori were overall judged as harder and heavier.

For Glavens, softness and weight judgments did vary less compared to geometric objects, however, judgments for bell peppers were about as variable as those for super-ellipsoids (Figure 9). This is the case even though bell peppers are comparable in terms of their local surface shapes and surface curvature magnitudes (Norman et al., 2012). This resonates well with a previous study showing better shape discrimination between bell peppers compared to between Glavens—presumably because of their more variable surface appearance (Norman et al., 2016). Here, Glavens were overall judged as rather soft and of medium weight whereas bell peppers were judged as somewhat harder and rather heavy. The differences in softness and weight ratings for bell peppers were not well explained by the differences in their curvature. Therefore, we speculate that higher-level cognitive inferences might have been involved (e.g., related to the extent to which some bell peppers seem to “defy gravity” by curling up from an imagined ground plane), but the current study cannot tease those apart. Note that this should be considered in future studies that use apparently neutral three-dimensional geometric objects such as Glavens or bell peppers to investigate material perception. Even though they might not be perceived as similar to particular objects, they will bias estimations of material properties.

Second, to investigate the effect of mesoscale local shape features, we defined classes of spiky (four classes), protrusion (two classes), layered (two classes), and bumpy (one class) transformation stimuli, varying in amplitude and frequency of their defining surface features. Among all of them, we find substantial variations of softness and weight judgments depending on the type, magnitude, and frequency of surface shape features (Figure 8). For example, with increasing spikiness of shape features, objects are generally judged as harder and heavier (Figures 6 and 7). For gilled objects, we find something similar: they are judged as harder and heavier with increasing amplitude and frequency of their layers (Supplementary Figures S2A–S2C). For warty objects (Supplementary Figures S1A–S1C), stimuli with very few warts are judged as softer and lighter. At the same time, the pattern of results is more complicated for the scaly (Supplementary Figures S2D–S2F), knobby (Supplementary Figures S1D–S1F) and bumpy objects (Supplementary Figure S3). For those stimuli, changes in judged softness and weight often do not follow the variation of the stimulus parameters but are rather step-wise with particular high or low ratings for specific combinations of amplitude and frequency (e.g., the scaly objects with the smallest frequency and amplitude of scales were judged as rather hard and heavy compared to all other scaly objects). When comparing those judgments of material properties to object curvature as a measure of surface variation, we find significant correlations between mean curvatures and softness and weight ratings. These correlations are substantially higher for a number of classes when evaluated within classes rather than across all stimuli (Figure 10)—in line with decisions about material properties that rely on the type of features (e.g., spikes vs. knobs), as well as on their expression (e.g., number and pointedness of spikes).

The decisive role of feature type for judgments of softness and weight is also evident when directly comparing the results for warty and knobby objects (Figures 2C and 2D). Both stimuli classes are very similar with respect to the number and frequency of their surface features, except that warts are more rounded than the angular knobs, resulting in opposite effects of feature frequency on softness and weight ratings (Supplementary Figures S1F and S3F) and reversed correlations with mean curvature (Figure 10A). Interestingly, bumpy objects with their overall smoother appearance fall somewhere in between (Figure 2G): their softness ratings show a similar pattern to those of the smooth warty objects whereas their weight ratings are similar to those of the more angular knobby objects (Supplementary Figure S3C and Figure 10A). At this point, we can only speculate about the source of these opposite effects of surface curvature on softness versus weight ratings in bumpy objects–for example, surface curvature might influence some other object property (e.g., perceived size) which in turn might affect weight judgments.

The reported effects of mesoscale local shape features on estimations of material properties are in line with previous results: For example, Giesel and Zaidi (2013) illustrated effects of mesoscale texture and local shape features on material property judgments for real-world images of different textiles. Specifically, they showed that judgments of roughness, thickness, and undulations vary with the contrast at different spatial frequencies (which can be used to reconstruct three-dimensional shape from images). Pinna and Deiana (2015) showed that observers associate local shape and megascale deformations of two-dimensional contours with particular material properties. Here, we demonstrated related effects of mesoscale local shape features on softness and weight judgments for three-dimensional objects that were specifically designed to cover a wide range of different types of surface transformations and their parametric variations.

Notably, we obtained these effects for static, unfamiliar objects that do not evoke reliable semantic associations (Figure 11). This is important because material properties might be inferred by recognizing learned image features of particular materials (e.g., folds and wrinkles suggest textiles) and then retrieving associated material properties from memory (e.g., textiles are soft and smooth rather than hard and rough) (Fleming, 2017; Schmidt et al., 2017; Schmidt, 2019; van Assen & Fleming, 2016). However, the lack of reliable semantic associations for our stimuli rather suggests that observers estimated material properties directly from image features (e.g., when a material looks like it would deform easily under pressure, it is soft rather than hard). Note that this estimation might also include the inferred causal history of an object (e.g., when an object looks as if it has been twisted or bent, it is soft rather than hard).

Of course, shape features at all scales can be used either to infer the depicted material identity via semantic association or to directly estimate material properties. However, our results of the naming experiment suggest that different judgments of softness and weight were not merely based on direct semantic associations between stimuli and particular real-world objects, materials, or transformations. Rather, it seems that mesoscale shape features were used for estimation such that particular local shape features suggest particular expressions of material properties.

Consequently, we suggest that local shape features be considered next to microscale and mesoscale texture and megascale shape features (Koenderink & van Doorn, 1996) when investigating effects of object shape on the visual estimation of material properties. Future studies should aim to develop models that relate these property estimations to objective measures of surface shape (Paulun et al., 2017) or to perceptual midlevel shape features (such as “blobbiness” or “symmetry”; van Assen et al., 2018). This will allow us to develop and test predictions for other sets of stimuli (e.g., images of real-world objects) and other types of materials. For example, it would be interesting to see whether human observers are using similar midlevel shape features (with different weighting) to estimate material properties of solid objects (e.g., softness) as they use to estimate properties of liquids (e.g., viscosity; van Assen et al., 2018). This would be in line with a general-purpose statistical appearance model where different materials and material properties are organized along manifolds in the same latent representational space (Fleming, 2014; Fleming, 2017; Fleming & Storrs, 2019).

## Supplementary Material

Supplement 1
