# Supplementary material for: Softness and weight from shape: Material properties inferred from local shape features
Source: J Vis. 2020 Jun 3;20(6):2. doi: 10.1167/jov.20.6.2 (PMC7416911; doi:10.1167/jov.20.6.2)
Supplement: Supplement 1 [file jovi-20-6-2_s001.docx]

**Supplementary Material**

**Figure S1**

Figure S1 shows rating results for warty (A-C) and knobby objects (D-F). Stimuli are plotted as a function of the two parameters defining the amplitude and frequency of the warts and knobs.


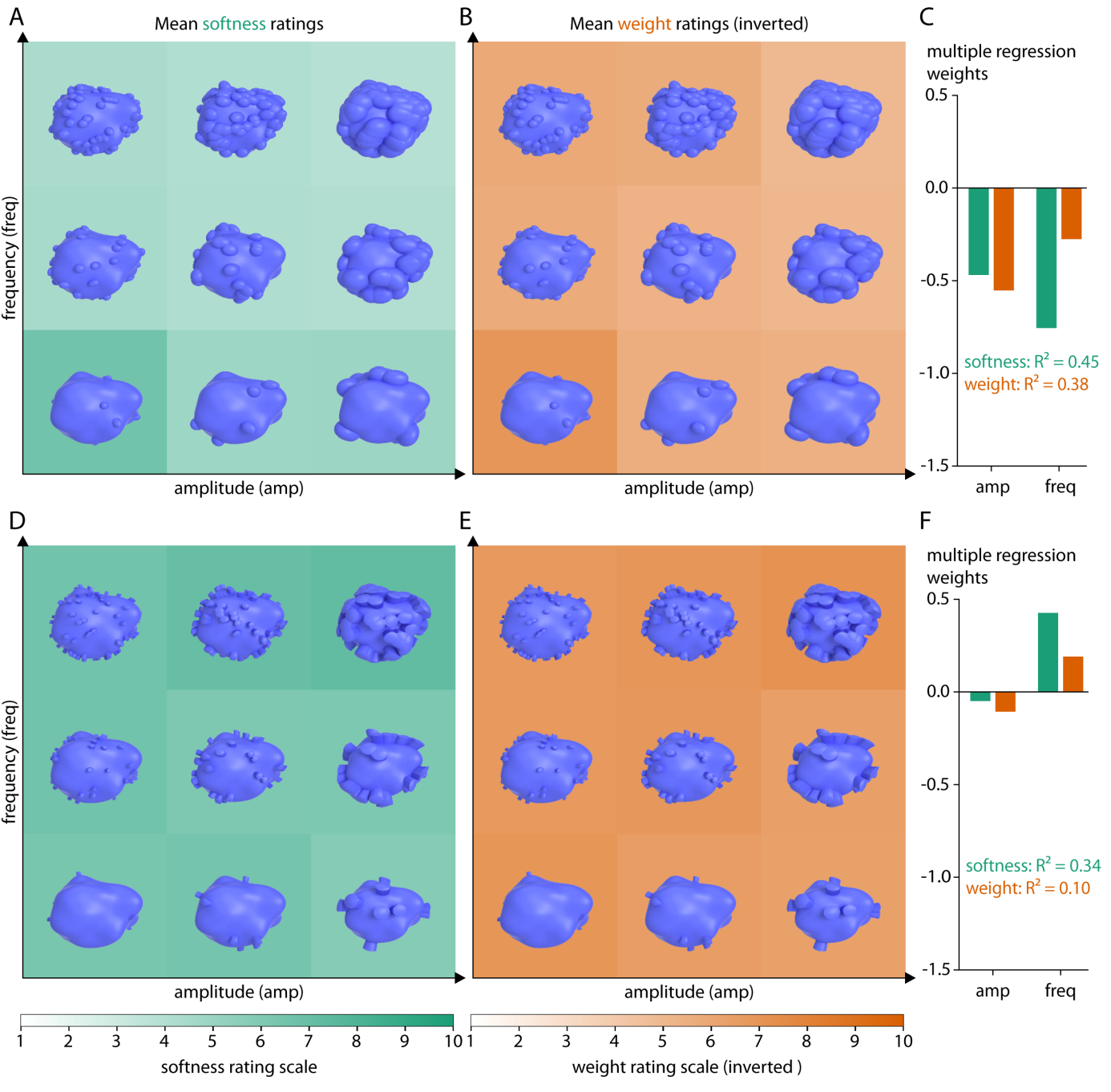


**Figure S1.** Rating results for the two classes of protrusion objects. (A, D) Softness ratings and (B, E) inverted weight ratings for warts and knobs, respectively, averaged across participants and the 5 base objects. More saturated green values correspond to softer objects, more saturated orange values correspond to lighter objects. Stimuli are plotted as a function of protrusion amplitude and frequency (see text for details). (C, F) Multiple regression fits (R^2^ values) and regression weights for amplitude and frequency on mean softness (green) or mean inverted weight (red) ratings.

Mean softness ratings of warty objects were about M = 4.0 (range = [2.4, 7.2]; SD = 1.10), mean weight ratings were about M = 5.1 (range = [3.9, 7.4]; SD = 0.83). Mean softness ratings of knobby objects were about M = 6.1 (range = [5.1, 7.4]; SD = 0.61), mean weight ratings were about M = 6.3 (range = [4.9, 7.3]; SD = 0.58). For warty objects, multiple regression analyses using wart amplitude and frequency as factors explained considerable variance for softness (R² = 0.45) or weight ratings (R² = 0.38). For knobby objects, multiple regression analyses using knob amplitude and frequency as factors did explain more variance in softness ratings (R² = 0.34) compared to weight ratings (R² = 0.10).

**Figure S2**

Figure S2 shows rating results for gilled (A-C) and scaly objects (D-F). Stimuli are plotted as a function of the two parameters defining the amplitude and frequency of the gills and scales. Mean softness ratings of gilled objects were about M = 5.6 (range = [4.3, 7.1]; SD = 0.74), mean weight ratings were about M = 5.9 (range = [4.7, 7.1]; SD = 0.61). Mean softness ratings of scaly objects were about M = 5.3 (range = [3.9, 6.3]; SD = 0.49), mean weight ratings were about M = 6.1 (range = [4.5, 7.2]; SD = 0.54). For gilled objects, multiple regression analyses using gill amplitude and frequency as factors did explain more variance for softness (R² = 0.36) than for weight ratings (R² = 0.20). For scaly objects, multiple regression analyses using scale amplitude and frequency as factors did hardly explain any variance in softness (R² = 0.07) and weight ratings (R² = 0.06).


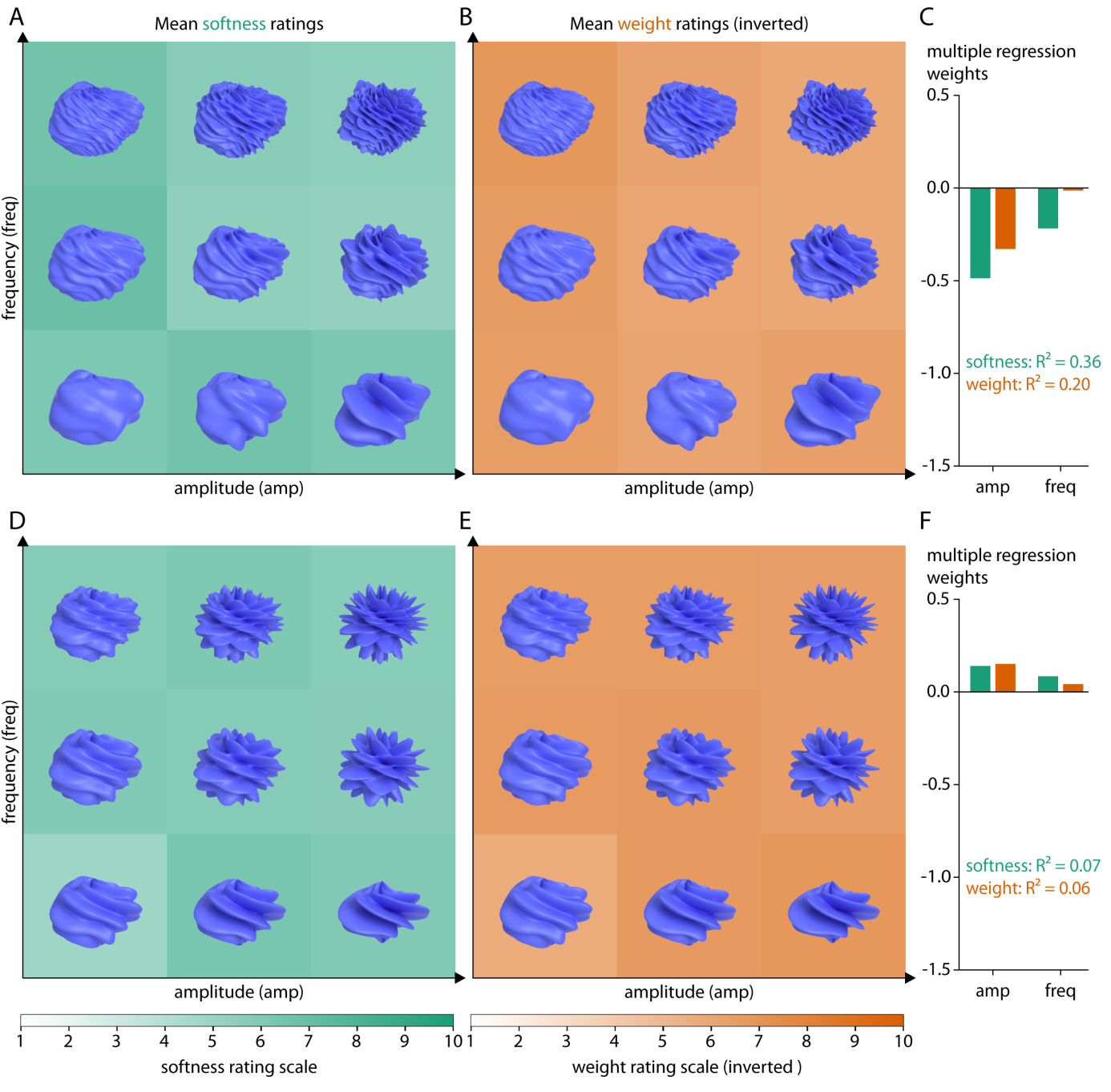


**Figure S2.** Rating results for the two classes of layered objects. (A, D) Softness ratings and (B, E) inverted weight ratings for gills and scales, respectively, averaged across participants and the 5 base objects. More saturated green values correspond to softer objects, more saturated orange values correspond to lighter objects. Stimuli are plotted as a function of layer amplitude and frequency (see text for details). (C, F) Multiple regression fits (R^2^ values) and regression weights for amplitude and frequency on mean softness (green) or mean inverted weight (red) ratings.

**Figure S3**

Figure S3 shows rating results for bumpy objects. Stimuli are plotted as a function of the two parameters defining the amplitude and frequency of bumps. Mean softness ratings of bumpy objects were about M = 6.1 (range = [5.3, 7.8]; SD = 0.50), mean weight ratings were about M = 6.5 (range = [5.2, 7.3]; SD = 0.54). Multiple regression analyses using bumpy amplitude and frequency as factors did explain somewhat more variance for softness (R² = 0.18) than for weight ratings (R² = 0.06).


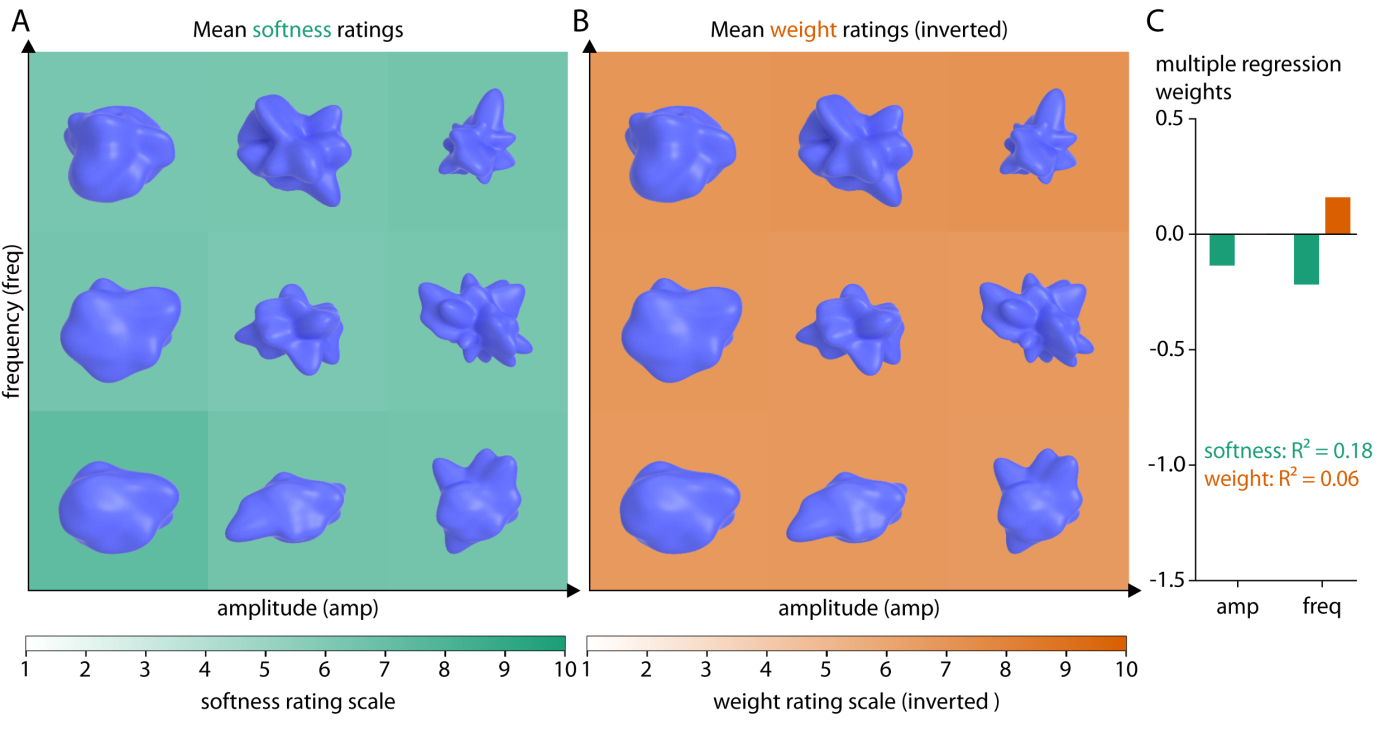


**Figure S3.** Rating results for the bumpy objects. (A) Softness ratings and (B) inverted weight ratings for bumps, averaged across participants and the 5 base objects. More saturated green values correspond to softer objects, more saturated orange values correspond to lighter objects. Stimuli are plotted as a function of bump amplitude and frequency (see text for details). (C) Multiple regression fits (R^2^ values) and regression weights for amplitude and frequency on mean softness (green) or mean inverted weight (red) ratings.

**Figure S4**


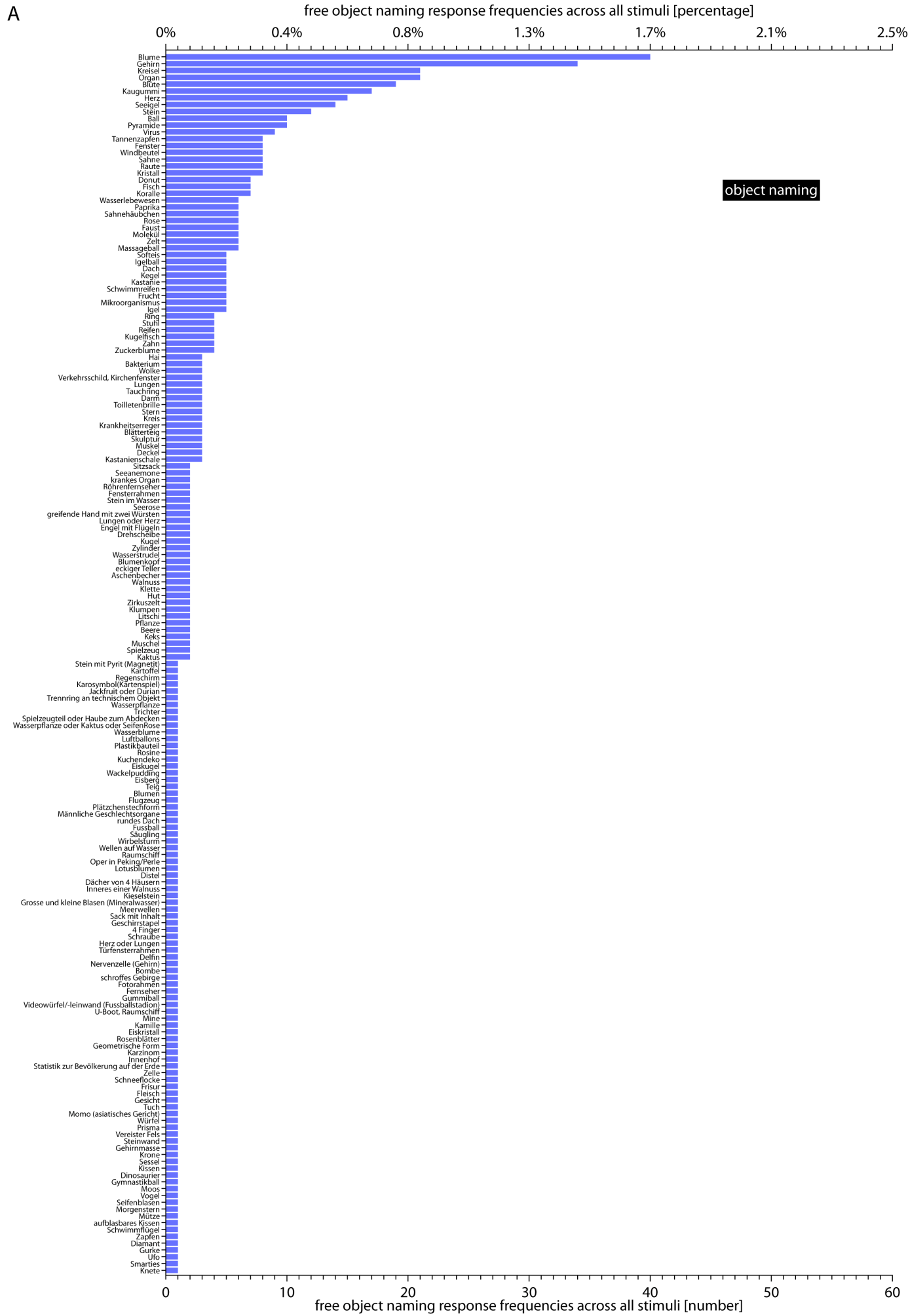


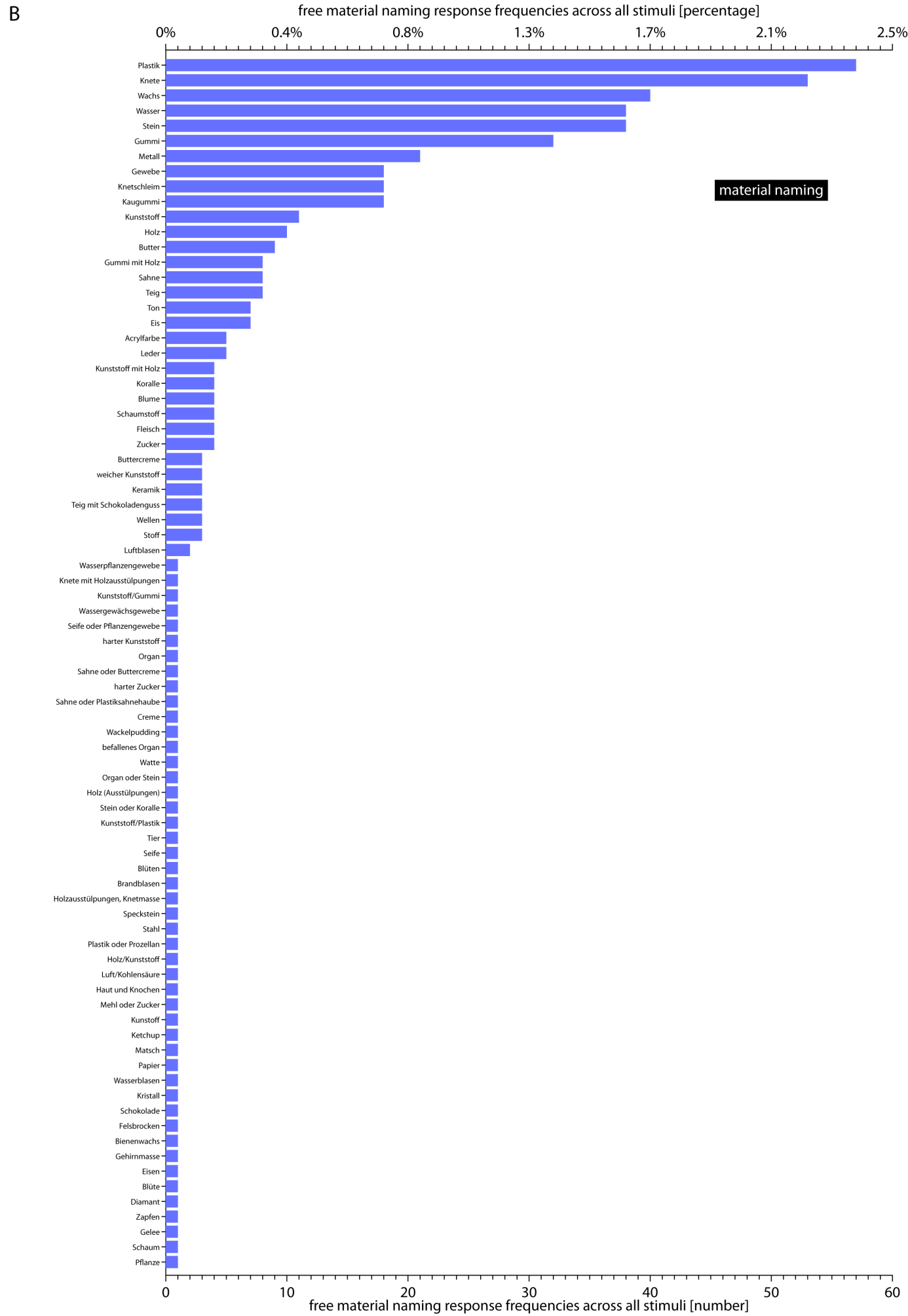


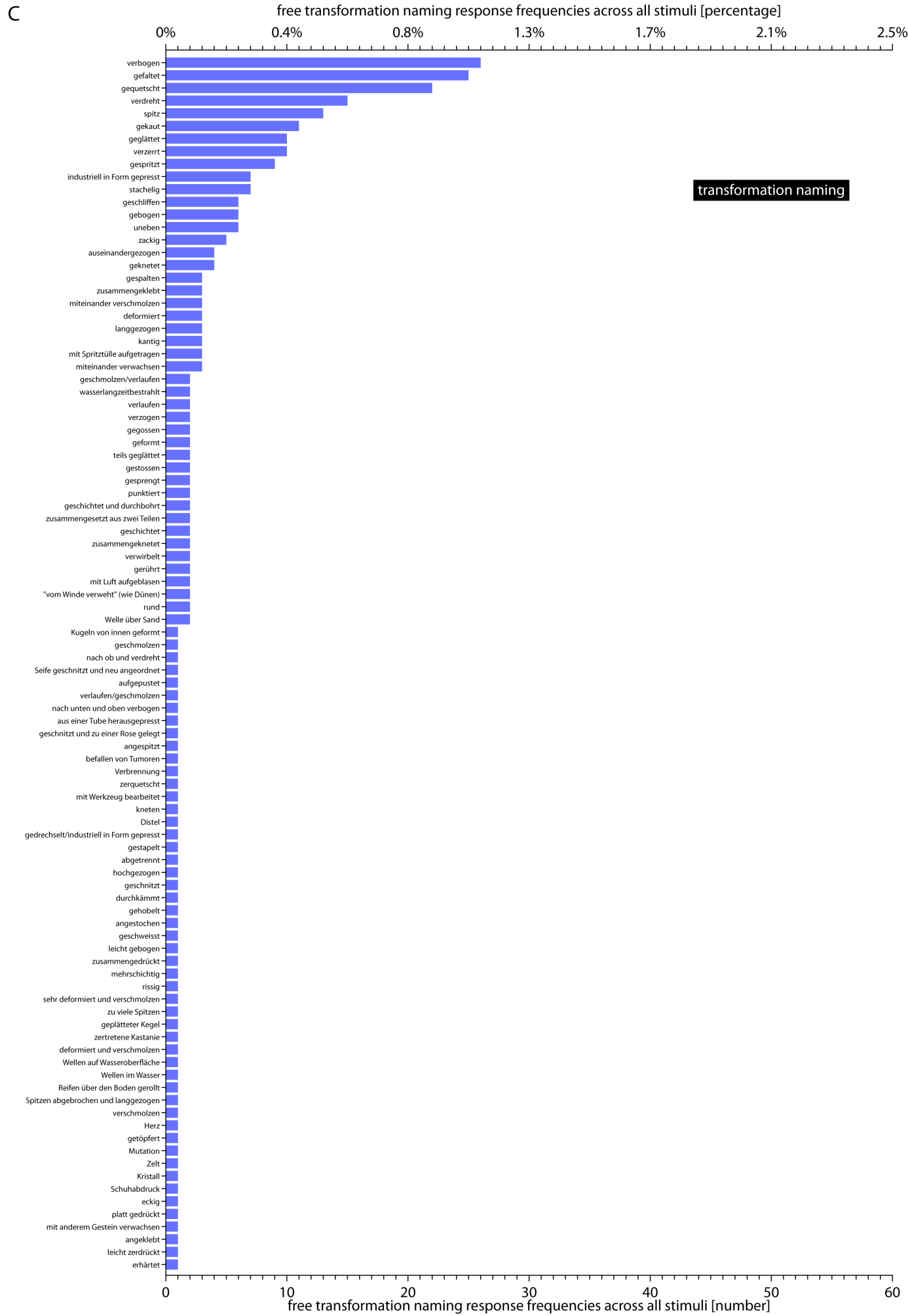


**Figure S4.** Frequencies of all responses for the free object naming task (raw number of responses on lower axis; percentage of all responses on upper axis−with 5 participants × 479 stimuli = 2395 responses) across all participants and stimuli for (A) objects, (B) materials, and (C) transformations.
